# Immune Dysregulation in Sepsis. A Narrative Review for the Clinicians

**DOI:** 10.3390/biomedicines13102386

**Published:** 2025-09-29

**Authors:** Asimina Valsamaki, Vasileios Vazgiourakis, Konstantinos Mantzarlis, Efstratios Manoulakas, Demosthenes Makris

**Affiliations:** Intensive Care Unit, Faculty of Medicine, University of Thessaly, 41110 Larissa, Greece; vasvazg@yahoo.com (V.V.); mantzk@outlook.com (K.M.); stratosfox@hotmail.com (E.M.); dimomakris@uth.gr (D.M.)

**Keywords:** sepsis, cytokine storm, inflammatory response, biomarkers, granulocyte-macrophage colony-stimulating factor (GM-CSF), T cell exhaustion, systemic inflammatory response syndrome (SIRS), compensatory anti-inflammatory response syndrome (CARS), miRNA, bacterial biofilms

## Abstract

Immune dysregulation presents a significant clinical challenge due to its rapid progression and complex interplay between hyperinflammatory and immunosuppressive responses. Different responses from the innate and adaptive immune systems can result in diseases such as immunoparalysis, cytokine storms, and secondary infections. Current diagnostic methods remain non-specific and time-consuming, delaying targeted interventions. A compartmentalized approach to immune monitoring, distinguishing innate and acquired immune response functional differentiation, is essential for distinguishing between hyperactivation and suppression. Key biomarkers, including cytokines, Granulocyte-Macrophage Colony-Stimulating Factor (GM-CSF), and CD4/CD8 counts, as well as Programmed Death Ligand-1 (PDL-1) and V-type immunoglobulin domain-containing suppressor of T cell activation (VISTA) regulators, can guide personalized treatment strategies. Although they need more clinical validation, novel therapeutic methods such as cytokine inhibitors, immunological stimulants, and immunomodulators have demonstrated promise. Early diagnosis and precision medicine developments could lead to better patient outcomes. Advances in non-coding RNAs have led to specific diagnostic panels based on microRNA (MiRNA) levels. A deeper understanding of immune imbalance in sepsis is critical for optimizing treatment and reducing mortality rates. This review highlights emerging diagnostic and therapeutic strategies to address the multifaceted nature of sepsis-related immune dysregulation.

## 1. Introduction

Microbe-induced immune dysregulation is practically one of the most frustrating situations for clinicians. This is due to several factors, which in combination orchestrate the detrimental effect of the early onset of sepsis within hours of the infection, limiting time for early diagnosis. The diverse nature of microbial infections elicits variable immune responses, and multiple tissues and organs are variably affected in the progression of sepsis. The multiple effects on immune modulation, ranging from immunosuppression and immunoparalysis to hyperactivation and inflammatory storm, reflect the variable input and variable effector responses of immune imbalance by the innate and adaptive systems. The patient’s own genetic and pathological background, along with comorbidities, confer additive susceptibility to secondary infections [1,2,3,4,5]. Therefore, a wide range of factors are implicated in the onset and the rate of progression of sepsis. At the same time, the existing applicable diagnostic means remain mostly non-specific and time-consuming. In essence, the clinicians are faced with trial and error and hit and run approaches. In contrast, hundreds of RCTs (Randomized Clinical Trials) have failed to uncover therapeutic benefits [6,7], concluding that effective treatments for sepsis are not available [5]. Epidemiologic evidence of sepsis-related clinical responses is the high rate of deaths, estimated to be 20–30% [8,9], and the significant negative quality of life effect on surviving patients [10].

Herein, immune dysregulations in response to sepsis, causing compensatory anti-inflammatory immune responses (CARSs), will be reviewed, and the effects of the loss of homeostatic equilibrium in immunomodulation will be described from a pathophysiological point of view, hoping to elicit a better understanding of the clinical challenge at hand. To this effect, a compartmentalized approach will be utilized to serve as a guide for quicker and more reliable treatment.

The main theme of the present study aims at distinguishing the differential immune responses per patient case and the necessary compartmentalization of these responses into innate and acquired. In this respect, recent advances in more specific and faster biomarkers are being discussed, and alternative therapeutic avenues are being proposed. The second theme of the study involves the concept that there is not simply one pathway leading to sepsis but rather an unpredictable onset of multiple molecular and cellular dysregulations, affecting any or several tissues. Therefore, a more personalized approach to diagnosis and treatment could offer significant benefits.

## 2. Materials and Methods

A comprehensive literature search was performed to identify relevant articles published in peer-reviewed journals. A systematic search of electronic databases, including PubMed, Scopus, Web of Science, and Google Scholar, was conducted to identify studies related to immune dysregulation in sepsis. Keywords used in the search included sepsis, immune dysregulation, cytokine storm, immunoparalysis, biomarkers, GM-CSF, T-cell exhaustion, SIRS (systemic inflammatory response syndrome), CARS (compensatory anti-inflammatory response syndrome), precision medicine, and antimicrobial resistance. Reference lists of selected articles were also reviewed to identify additional relevant studies.

## 3. Immune Dysregulation in Sepsis

According to Morris et al. (2022) [7], two useful elements in sepsis therapeutics are the elucidation of elicited immune responses as active or suppressed and as blood- or tissue-inflicted. This observation leads to the concept of compartmentalization of immune responses. When signs of immune hyperactivation in both blood and tissue are evident, immunosuppression should have a positive impact. On the other hand, if immunosuppression is observed in both tested compartments (concordance of immune responses), immunostimulants will benefit. However, in cases of discordant immune state, that is, immune activation detected in blood and immune suppression in tissues, or vice versa, the therapeutic response becomes uncertain. In these cases, further diagnostic investigation is required to ascertain immune pathways over- or under-activated in sepsis. In other words, blood testing of known biomarkers may not lead to an accurate diagnosis and implementation of treatment. An example of such a compartmentalized approach is the granulocyte colony-stimulating factor (GM-CSF), with a dual role in sepsis-inflicted lung tissue. Although GM-CSF is required both for normal lung alveoli homeostasis and host defense to infections, in pathologic conditions, its aberrant expression in diseased tissue may drive excessive inflammation and damage [11].

Immune response to infection involves either or both innate and acquired immune mechanisms. Dysregulation of one does not necessarily imply malfunctioning of the other. Further, the inflammatory hyperactivation of one does not always provoke enhanced activity of the second. Innate immune responses are mainly based on PRRs (Pattern Recognition Receptors), recognition of PAMPs (pathogen-associated molecular patterns), and extended phagocytic activity by the macrophages [12,13]. The defensive overreaction of this response leads to the “cytokine storm” and the extensive release of IL-6, Il-10, IFNγ, INFα, and other inflammatory factors, which ultimately and very shortly lead to septic shock, accompanied by the symptoms of high fever and tissue and/or organ damage, while usually, a decrease in phagocytosis occurs due to extensive macrophage exhaustion [4,14,15].

Although not universal, one proposed mechanism for the cytokine storm includes a feedback stepwise loop.

- Upon sepsis onset, macrophage TLRs (toll-like receptors) recognize and bind microbial PAMPs (pathogen-associated molecular patterns), such as LPS, in cases of Gram-negative pathogens. TLR engagement promotes the activation of intracellular signal transduction pathways, such as, among others, NF-kB and MAPK pathways, leading to the release of cytokines as a defense mechanism [16].
- Neutrophil activation is observed. In this activation, aside from traditional mechanisms (release of cytokines, phagocytosis, reactive oxygen species), neutrophils release extracellular traps (NETs), targeting bacteria clearance in the circulation under normal conditions, but the excessive release of NETs under neutrophil hyperactivation leads to the shift of endothelial cells toward a pro-inflammatory phenotype, a degradation of endothelial glycocalyx, and an increase in endothelial permeability. These consequences have the result of dramatic endothelial dysfunction with increased vascular permeability, tissue hypoperfusion, and microcirculatory flow disturbance, findings that characterize late sepsis and, importantly, organ failure [17].
- Natural Killer (NK) cells, as one of the most important lymphocyte cell types of innate immune responses, can orchestrate early responses to bacterial pathogens. Their role in amplifying responses of myeloid cells, especially macrophages, is generally thought to be mediated by the production of IFN-γ. Their excessive activation and IFN-γ production lead to systemic inflammatory response during sepsis and organ dysfunction, although in bacterial sepsis, their role derives basically from studies in mice. Human studies have shown, until now, a correlation between NK function and outcomes, so their exact role in human bacterial infections still remains to be more clearly defined [18].

Adaptive immune responses (AIRs) are based on the recognition of infected cells and the cumulative action of T and B lymphocytes, aiming at the neutralization of invading microorganisms and apoptosis of infected cells [19]. In a septic state, the AIR system is usually observed in a state of suppression, as evidenced by T cell apoptosis and decreased CD4 and CD8 counts [4]. Afterward, when the counteractivity state of the two immune systems occurs, clinically, a distinction should be made, utilizing suppressors for one system and stimulants for the second. Further, the crucial point of time, zero, which represents the transition from infection to sepsis, as determined by SOFA (Sequential Organ Failure Assessment) and EWS (Early Warning Score), is essential [4]. Levels of cytokines, contrasted to CD4/CD8 counts, could determine the active state of either system or direct treatment accordingly.

The immune response in sepsis is characterized by a dual nature, comprising two distinct but interrelated pathological states: (1) excessive overreaction, manifesting as hyperinflammation, and (2) suppression via immune exhaustion, often termed immunoparalysis. These opposing states of immune dysregulation necessitate separate examination. Differentiated biomarkers for innate and adaptive immunity can guide clinical interventions by tailoring therapeutic strategies to the patient’s unique immunological profile. A personalized clinical approach, emphasizing individualized immunotherapy based on immune phenotyping, is strongly recommended to restore immune homeostasis, as suggested by Cao et al. [5]. The primary focus should remain on identifying which pathway of the immune response exhibits imbalanced activity, with appropriate biomarkers and indicators for either hyperactive or suppressed states of immune function outlined in Table 1.

The “cytokine storm,” a hallmark of exaggerated innate immune responses, is fueled by the overproduction of pro-inflammatory cytokines and reactive oxygen species (ROS). This hyperactivation damages endothelial tissue, resulting in increased vascular permeability and glycocalyx distortion [4]. Consequently, disruption of homeostasis between coagulation and fibrinolysis ensues, favoring a pro-thrombotic state mediated by excessive thrombin generation [25]. Additionally, the innate immune response activates neutrophils, promoting the formation of neutrophil extracellular traps (NETs) that aid in microbial containment. However, excessive NET formation exacerbates thrombotic complications [20]. This intricate interplay between endothelial cells, coagulation pathways, and immune responses is now recognized as a pivotal pathophysiological mechanism driving bacterial sepsis, ultimately contributing to multi-organ failure [26]. This recognition underscores its potential as a target for innovative therapeutic strategies.

Another critical immune defense mechanism, the complement system, also suffers from dysregulation during sepsis. The excessive generation of complement factors, particularly C3a and C5a, signals a pro-inflammatory response [21]. These factors are implicated in neurodegenerative processes via their influence on neurotrophils, which may play a role in septic encephalopathy pathogenesis as well [27,28]. Additionally, C5a excess in the later stages of sepsis disrupts neutrophil function, diminishing bactericidal activity and increasing susceptibility to infections [29] (Yan & Gao 2012). This dysregulation accelerates sepsis progression, making C5a a reliable biomarker for severe sepsis [21]. Complement dysregulation extends its pathological impact by influencing coagulation pathways, further contributing to the pro-thrombotic state observed in sepsis, as recently reviewed by Arora et al. (2023) [4].

To address these interconnected mechanisms, a comprehensive approach is essential. This includes the identification of key biomarkers to monitor immune activity, the use of targeted therapies to modulate excessive inflammatory responses, and interventions to restore immune balance. Advances in understanding the dynamic interplay between immune pathways and coagulation systems open the door to novel therapeutic targets, which hold promise for improving outcomes in septic patients.

### Diagnosis and Treatment Avenues for Sepsis-Related Immune Alterations

From time zero, sepsis follows an exponential progression when the described multi-response system is dysregulated, resulting in systemic expression of the infection. Effective therapeutic avenues cannot be defined before the per-patient case deviations of the defense mechanisms are explored. Therefore, a timely and correct diagnosis is essential for an effective treatment strategy. The fact is that current conventional diagnostic approaches are neither quick nor precise. As argued above, a whole set of biomarkers with critical function in sepsis stages could provide increased specificity as to per-case immune dysregulation, thus orienting the clinician into appropriate therapeutic responses. These biomarkers, such as GM-CSF, IL-6, and other cytokines (IL-1, IL-3, and TNFa), complement C5a, macrophage, neutrophil, and lymphocyte CD4-CD8 counts, among others, and are summarized in Table 1. Since there is a constant interplay between innate and acquired immune system dysregulation in sepsis, in this table, the proposed biomarkers are categorized in terms of their function rather than the system affected.

## 4. Non-Coding miRNA as Biomarkers

Early diagnosis of the type of infection could be possible via the utilization of MIR panels. Recent extensive reviews on the role and predictive significance of non-coding RNA molecules emphasize their importance as sepsis biomarkers [3,30]. MIRs, indicating the type of infection about sepsis and/or exhibiting expression levels indicative of sepsis, are summarized in Table 2.

## 5. Antibodies as Useful Treatment Tools

IL and other cytokine inhibitors, mainly monoclonal antibodies and hybrids thereof, have been tested in animal models and clinical studies, not only in the prevention of the cytokine storm but at later stages of sepsis progression. IL-1 has been a primary therapeutic target in recent trials. Anakinra (rIL-1ra) has been tested in several studies and has shown mainly positive results in specific cases. In an early study, septic patients treated with anakinra had increased survival time according to Knaus et al. (1996) [39]. Patients with HBD/DIC (hepatobiliary dysfunction and disseminated intravascular coagulation) showed a significant increase in survival time [40]. In a recent report [41], anakinra was shown to reduce severe respiratory failure, while it restored the balance between pro- and anti-inflammatory mechanisms in a suPAR-based (soluble urokinase plasminogen activator receptor) study. SOFA scores indicated increased survival in about 50% of patients with MALS (macrophage activation-like syndrome) [42]. Antibodies against IL-3 also showed promising results in septic animal models since reduced organ failure and increased survival were observed [43]. Yet, it should be kept in mind that IL-3 studies indicate positive outcomes in viral infections, such as pneumonia [44]. Other studies oriented towards a blockade of cytokines (IL-6 and TNFa) have also produced partially positive results in animal models or pre-clinical trials [5].

## 6. Cytokine Administration in Treating Specific Septic Conditions

During sepsis, the immune system and specific cytokine release are not always exaggerated, but in cases where immunosuppression is observed, several studies have utilized the administration of specific cytokines to boost immune responses. As mentioned earlier in this study, a strong indication of the suppression of acquired immune response is the low counts of CD4+ and CD8+ lymphocytes. IL-7 levels have also been observed to be lower in sepsis [45]. Indeed, several studies indicate strong positive results with IL-7 administration, including the reversal of CD4-CD8 counts and rhIL-7 reversed T lymphocyte depletion in severe COVID-19 infections [46,47,48]. Endogenous IFN-γ is one of the cytokines responsible for the cytokine shock observed in sepsis. Yet, when immunosuppression of the acquired immune system is observed, externally administered IFN-γ ameliorated immunoparalysis [49] and partially re-instated immune activity in fungal infection-based sepsis [50]. In a more recent multicenter study on sepsis-induced immunosuppression, IFN-γ restored immune activity [51].

## 7. Therapeutic Effects of Modulation of Sepsis-Related Regulatory Factors

Beyond cytokine inhibitors and activators, several other factors have been shown with clinically useful immune-modulatory activity. As discussed earlier, GM-CSF plays an important dual role in immune regulation. This dual role has been investigated in trials, supporting the notion that GM-CSF is a valuable tool in immunosuppressive status due to sepsis by reversing disease severity, as marked by mHLA-DR expression enhancement and cytokine release [5,52], even though it may not be offered in recovery at the stage of severe sepsis [53]. The immune checkpoint neutrophil factor programmed cell death protein 1 (PD-1) is directly associated with sepsis-produced immunoparalysis. Agents blocking PD activity have shown encouraging results in restoring a balanced immune response during sepsis. Anti-PD–1 antibody reversed T lymphopenia [24] and restored immune activity [54]. FDA-approved monoclonal anti-PDl–1 antibody nivolumab has been used successfully for the treatment of septic shock conditions, and it has been proven safe and is tolerated [55]. Based on the above, GM-CSF inhibitors may be of clinical value in the early stages of sepsis, while immune checkpoint blockers may be utilized for late sepsis. Adjuvant therapy in septic mice, utilizing PD-1 and CTLA-4 antibodies (cytotoxic T lymphocyte-associated protein 4), reversed immunosuppression [56]. The CTLA-4 factor is expressed in CD4, CD8, and Treg lymphocytes, and its effects seem to be in a dose-dependent manner since the overexpression downregulates acquired immune response and lower levels activate it [22,57]. Therefore, CTLA-4 levels could define acquired immunity status in sepsis.

It becomes obvious from the above paradigms that the same molecular factors may produce different effects, depending on the levels of expression, or act differently, depending on the sepsis stage of progression and the type of immune response dysregulated. Further, it has been shown that immune checkpoint-related proteins, such as CTLA-4 and VISTA, may have their function determined or modulated, by which gene variants are expressed during sepsis [58,59], a molecular event that raises the possibility of genomic analysis, contributing to predicting the type and kind of immune dysregulation. VISTA (V-domain IG suppressor of T cell activation) has been shown to downregulate the activation of T lymphocytes. The inhibition of VISTA with a high-affinity monoclonal reduced T cell apoptosis and cytokine release and enhanced microbial clearance [23]. Yet, on the contrary, it has also been shown that VISTA acts positively on acquired immune responses during sepsis, regulating T-reg expression and reducing disease mortality and morbidity [60], as well as affecting macrophages in attenuating inflammation [61]. The above further supports the dual activity of a single factor, and genetic variant analysis could be a useful tool in directing treatment to up- or downregulate a checkpoint modulatory factor.

## 8. Utilization of Exogenous Immunoglobulins

Treatment with the exogenous administration of immunoglobulins initially showed encouraging clinical results, following the notion that a quantitative deficit of immunoglobulins is directly linked with severity and mortality in sepsis [62,63,64]. Yet, since 2021, the guidelines of the Surviving Sepsis Campaign have raised doubts about the administration of exogenous immunoglobulins due to a lack of concrete evidence, even though this suggestion is based on a 2007 SBITS report [65].

## 9. Treatment with Corticosteroids

Corticosteroids, and especially glucocorticoids, have been one of the major and long-lasting treatment approaches. Earlier studies strongly suggested the use of steroid-type endocrine factors for the treatment of severe sepsis. This approach is based on the modulating effect of the hypothalamic–pituitary and adrenal neuroendocrine axis on the immune system dysregulation and inflammatory immune response during sepsis. Early clinical evidence indicated a significant effect on sepsis patients in terms of ICU length of stay as well as recovery from septic shock, with emphasis on tissue recovery [66]. The laboratory and clinical evidence showed total blockade of the pro-inflammatory NF-kB pathway [67]. Yet, as discussed above, the blockade of pro-inflammatory pathways is only part of the story, and sometimes activation is needed when immunoparalysis is observed in sepsis.

A very recent meta-analysis [68] concludes that corticosteroids can reduce mortality and increase the reversal of septic shock, yet they can present an increased risk for a multitude of side effects, such as neuromuscular deficiencies and hyperglycemia, affecting the quality of life of the recovered. At any rate, the use of corticosteroid substances is suggested as a last resort during latent sepsis stages. Annane et al. (2018) [69] used a combination of hydrocortisone and fludrocortisone to significantly reduce the 90-day mortality rate in patients with septic shock. On the contrary, in another trial, glucocorticoids alone or in combination with adjuvants with vitamins B1 and C showed no significant recovery from septic shock [70]. Given the controversy over corticosteroid treatment, Cao (2023) [5] rightfully suggests that specific endocrine phenotypes determined by genome-wide profiling could benefit from the specific therapy on a personalized precision medicine approach.

A summary of potential therapeutic means is presented in Table 3.

## 10. Discussion

Herein, a compartmentalization of immune responses during sepsis was discussed. It becomes obvious that immune responses in pathological states are not unified [7]. Immune response during sepsis may differ between anatomical sites of the body and indeed can be confined to a single compartment without affecting other sites of the body. Overactivation of the initial innate response may produce inactivation of the latent acquired response. Excessive innate response leads to cytokine storm, which may result in monocyte exhaustion. Since these cells can act as antigen presenters to T lymphocytes, their depletion leads to limited acquired immune response activity, as easily detected with CD4–CD8 counts. In such a case, both cytokine inhibitors, e.g., anakinra and T and B cell activators, may be needed. Anti-PD-1 monoclonal nivolumab has been shown to enhance immune response. Anti-CTLA–4 also enhanced AIR response. Lowering levels of initial cytokine release may effectively balance the global immune response. On the contrary, a lower initial innate response is boosted by exogenous ILs. The AIR system has been observed to be suppressed in conditions, such as cachexia, SIRS (systemic inflammatory response syndrome), CARS (compensatory anti-inflammatory response syndrome), or mixed anti-inflammatory response syndrome (MARS), which are all linked with the excessive release of pro-inflammatory cytokines. Monoclonals against TNF-α, IL-6, and IFN-γ, as well as thalidomide, have been shown to effectively ameliorate excessive inflammatory response [76].

As discussed earlier, Morris (2022) [7], who has proposed the compartmentalization concept for sepsis, observed that the immune response in the circulatory system may differ from tissue response. This perception leads to B cell inactivation in the circulatory system, as compared to the T lymphocyte activity in tissues. In such a case as B cell nonresponse, a boost with exogenous immunoglobulins may reverse the passive state and explain why sometimes, but not always, the administration of immunoglobulins is effective. Since the initial innate immune response (IIS) is non-specific and simply recognizes pathogen-associated molecular patterns (PAMPs) and does not retain the memory of the infection, a memory containing (via Th and B cells) AIR activation is an absolute requirement to confront infection prevalence and recurrence.

It has also been noted that complement factors, such as C5a overactivation, may lead to systemic immune dysregulation. Therefore, monoclonal inhibitors for these complement factors have the potential to restore partial immune response homeostasis. Furthermore, certain modulatory factors, such as GM-CSF, CTLA-4, and VISTA, depending on the level of expression, may produce different overall outcomes of response. A differentiated approach, depending on the level of expression, may require activation or inhibition to restore systemic homeostasis. Furthermore, as has been emphasized by Marshall (2018) [77], endothelial cells are also strong antigen presenters via the release of ICAM-1 immunoglobulin-like factor and are essential for monitoring systemic response or over-response.

It has become evident that an effective immune response is not so much a quantitative answer but rather a balanced response of the multiple components of the response, and differential diagnostic and therapeutic means should be used per occasion.

## 11. Conclusions

Sepsis presents a clinical headache for physicians, to say the least. The concurrent, concomitant, and comorbid effects due to simultaneous immune system dysregulations are difficult to prognosis, diagnose, and treat given the short time intervals between infection and time zero and rapid septic progression. The actual time point when the infection switches to cytokine storms and septic reactions will probably be the cornerstone of sepsis therapy in the future. Immune function switch at that point may be the crucial step for targeted therapies. This review was an attempt to compartmentalize the multiple immune imbalances during sepsis, in parallel with distinguishing useful factors during the stages of the disease process, to provoke new diagnosis and treatment strategies. In this spirit, several diagnostic markers and treatment techniques have been contrasted and evaluated. New concepts, such as miRNA screening, as early reliable tests and microbial biofilm formation, enhancing microbial resistance, were lightly introduced. It should also be noted that the new generation of biomarkers discussed herein also serves as therapeutic targets. In other words, the early detection of a biomarker may reveal the primary cause of disease, or even the disease progression stage, and guide therapeutic means accordingly.

The aftermath of this study, by recent and current philosophy on the subject, promotes a more personalized and precise approach to detecting and treating sepsis based on the separation of immune activation and immunosuppression mechanisms that may simultaneously occur, the distinction between innate and acquired immune system dysregulation, and the stage of disease progression.

## Figures and Tables

**Table 1:** Possible effective biomarkers are discussed extensively in the present study. Cell counts can be indicative of specific, specialized cell deficiency, whereas chemotactic and regulatory factors indicate both innate and acquired immune system deficiencies.

Biomarker	Involvement in Immune Response Mechanisms	Reference
**Cell count**		
CD4 and CD8	A strong indication of the acquired immunity system status of activation or inhibition is the drastic decrease in counts of both markers in the suppression state	Arora 2023 [4]
Macrophages	Exhaustion leads to apoptosis and immune depression	Zhang 2023 [17]
Neutrophils	Indicative of NETosis and possible inflammation of epithelial tissue	Zhang 2023 [17], Gould 2014 [20]
NKs	Excess activity reinforces cytokine storm	Guo 2018 [18]
**Chemotactic**		
IL-6	A strong indicator of innate immunity is excessive activity, cytokine storm, and septic shock	Doganyigit 2022 [14]
TNFa	A strong indicator of innate immunity is excessive activity, cytokine storm, and septic shock	
C3a and C5a	Strong indicators of pro-inflammatory response; C5a in excess in the late sepsis stage	Xu 2017 [21]
**Regulatory**		
GM-CSF	Driver of immune disease at excessive levels	Lang 2020 [11]
CTLA4	Overexpression downregulates the acquired immune response, and lower levels activate it	Washburn 2019 [22]
VISTA	Indicates T cell suppression	Tao 2021 [23]
PDL-1	Directly associated with sepsis-produced immunoparalysis	Patil 2018 [24]

**Table 2:** Non-coding functional RNAs have been associated with target-specific regulation of protein expression at the post-transcriptional level. Specific microRNAs (MIRS) that have been shown to modulate defense mechanisms in sepsis are listed below.

MIR	Sepsis-Related Functions	Reference
MIR-223-5p	Indicator of lymphocyte apoptosis in septic patients and has been shown to suppress the formation of inflammasomes when expressed in mice	Liu 2020 [31], Li 2022 [32]
MIR-155	Upregulated in myocardium and plasma in human sepsis	Vasques-Nóvoa F 2018 [33]
MIR-574-5p	The modulator of STAT activity is found to increase in the serum of septic patients.	Liu 2020 [31]
MIRS 150 and 143	Both miRs were downregulated in sepsis in correlation with SOFA scores, as found in purified T cells	Mohnle 2018 [34]
MIR-27a-6p	Sepsis progression rate indicator in sepsis-induced lung injury	Lu 2022 [35]
MIR-331	Downregulates CLDN2 activity and restores the cellular function of endothelial cells, as detected in the peripheral blood of septic patients	Kong 2020 [36]
MIR-147b	Degrades ADAM15 mRNA as a protective mechanism, acting in human vascular endothelial cells	Chatterjee 2014 [37]
MIR-96	Downregulated in sepsis produced by Gram (−) bacteria, as found in plasma fractions	Chen 2014 [38]
MIR-101	Downregulated in sepsis produced by Gram (+) bacteria, as found in plasma fractions	Chen 2014 [38]

**Table 3:** Therapeutic avenues based on compartmentalization of immune dysregulations involved in sepsis. All pertinent sources are included in the appropriate text section.

Type	Specificity	Effects	Therapeutic Effect
**Cytokine inhibitors** **(immunosuppressive)**	Anti-IL1 (anakinra)	Increase in survival time in patients with HBD/DIC Reduced severe respiratory failure while restoring the balance between pro- and anti-inflammatory mechanisms SOFA scores indicated increased survival in about 50% of patients with MALS (macrophage activation-like syndrome)	Absolute survival benefit (20–50%) [42]
	a-IL-3	Showed promising results in septic animal models since reduced organ failure and increased survival were observed	
**Cytokine boosters** **(Immunostimulants)**	IL-7	Boosts CD4 and CD8 T cell activation, reverses T cell depletion in severe COVID-19 immunoparalysis	Large immunologic effect (200–400% increases in lymphocytes), but not yet translated reliably into a clear, consistent, mortality benefit [71]
	IFN-γ	Amelioration of immunoparalysis	
**Key regulatory factors modulators** **(immonostimulants)**	a-PD monoclonals (nivolumab)	FDA-approved monoclonal anti-PDl–1 antibody nivolumab has been used successfully for the treatment of septic shock conditions, and it was proven to be safe and tolerated Reversal of T lymphopenia and restored immune activity	30–60% improvement in immune function markers—% of mortality not proven yet [55]
	GM-CSF administration or inhibitors	GM-CSF is a valuable tool in immunosuppressive status due to early sepsis by reversing disease severity, as marked by mHLA-DR expression enhancement and cytokine release GM-CSF excessive activity, leading to overactivation of the immune response, can be blocked, reversing its inflammatory action	Very strong biomarker effects (80–100%), but no proven survival benefit in large RCTs [72]
	CTLA-4 inhibitors	Appropriate modulation of CTLA-4 can either up- or downregulate lymphocyte response, and CTLA-4 levels could define acquired immunity	Strong biomarker effects—no definite clinical mortality effect yet [73]
	VISTA inhibitors and enhancers	Inhibition of overexpressed VISTA with a high-affinity monoclonal reduced T cell apoptosis and cytokine release and enhanced microbial clearance Enhancement of VISTA, when poorly expressed, acts positively on acquired immune responses during sepsis, regulating T-reg expression and reducing disease mortality and morbidity	Modulating VISTA has promising pre-clinical effects on sepsis—clinical data are essentially absent [74]
**Immunoglobulin enrichment** **(immunomodulatory therapy—can produce both immunostimulatory and immunosuppressive effects)**	IgM enrichment	Has shown encouraging clinical results by the notion that the quantitative deficit of immunoglobulins is directly linked with severity and mortality in sepsis	Relative reduction in mortality of 35–45% [75]
**Corticosteroids** **(immunomodulatory therapy—can produce both immunostimulatory and immunosuppressive effects)**		Significant effect on sepsis patients in terms of ICU length of stay as well as recovery from septic shock, with emphasis on tissue recovery and blockade of the NF-Kb pathway A combination of hydrocortisone and fludrocortisone significantly reduced the 90-day mortality rate in patients with septic shock Corticosteroids can reduce mortality and increase reversal of septic shock, yet present an increased risk for a multitude of side effects	5–12% mortality reduction, 20–25% faster resolution of shock [68]

## Data Availability

No new data was created.
